# How does medication-related osteonecrosis of the jaw (MRONJ) influence the health-related quality of life after surgery?

**DOI:** 10.1186/s12903-025-06171-3

**Published:** 2025-06-21

**Authors:** Oliver Bissinger, Josefa Greiser, Elisabeth Maier, Philipp Ehrmann, Tamara Kakoschke, Klaus-Dietrich Wolff, Carolin Götz

**Affiliations:** 1 Department of Oral and Maxillofacial Surgery, Medical University of Innsbruck, Anichstr. 35, Innsbruck, 6020 Austria; 2 Department of Oral and Maxillofacial Surgery, School of Medicine, Technical University of Munich, Klinikum Rechts der Isar, Ismaninger Str. 22, 81675 Munich, Germany; 3 Department of Oral and Maxillofacial Surgery and Facial Plastic Surgery, University Hospital LMU Munich. Lindwurmstr. 2A, 80337 Munich, Germany

**Keywords:** Bisphosphonates, Antiresorptive agents, Antiangiogenic agents, MRONJ, Quality of life, Awareness, Postoperative outcome

## Abstract

**Background:**

Medication-Related Osteonecrosis of the Jaw (MRONJ), an established side effect of antiresorptive and antiangiogenic medication, could sometimes require surgical intervention, such as decortication. The objectives of this retrospective patient survey study were to evaluate the effect of surgical treated MRONJ on health-related quality of life (QoL) and to assess the awareness of the risk of MRONJ.

**Methods:**

The MRONJ-related QoL in both cancer and osteoporosis patients was determined in four different time periods after surgery applying the European Organisation for Research and Treatment of Cancer (EORTC) Quality of Life Core Questionnaire (QLQ-C30), a modified version of the Module for Head and Neck Cancer (H&N35) and MRONJ-specific questions. In addition the patients´ awareness of the risk of MRONJ when being prescribed the medication was examined. 65 patients with MRONJ (28 males and 37 females) who underwent surgery of the jaw were included in this study.

**Results:**

In comparison to normative data of the German population cancer and osteoporosis patients showed a reduction in their QoL in general. It was demonstrated that MRONJ causes an even further negative impact, particularly on role and social function. Amongst the MRONJ patients certain factors such as female sex, breast cancer as underlying disease, hypothyroidism, denture, nicotine abuse and multiple surgeries of the MRONJ were associated with worse QoL. The majority of patients suffered from a lack of knowledge regarding the risk of MRONJ when starting antiresorptive or antiangiogenic treatment. Unaware patients manifested worse QoL results and had to undergo more operations.

**Conclusion:**

QoL of MRONJ patients is deteriorated and the number of surgeries and the patients´ awareness have a significant impact on QoL. In order to reach better awareness amongst both patients, doctors and other health care professionals and to detect MRONJ at an earlier stage, the H&N35 and MRONJ specific questions should be routinely administered to osteoporosis and cancer patients when starting antiresorptive or antiangiogenic treatment.

## Background

Medication-related osteonecrosis of the jaw (MRONJ) is common significant side effect described for patients treated with antiresorptive agents (AR) or antiangiogenic agents (AA) [Physicians’ awareness of medication-related osteonecrosis of the jaw in patients with osteoporosis [1]. AR associated with MRONJ principally include bisphosphonates and denosumab. These medications play an essential role in the management of bone-resorptive oncological pathologies (e.g. multiple myeloma and skeletal metastases) and in the long-term treatment of dysmetabolic bone diseases (e.g. osteopenia and osteoporosis) [2, 3]. Application of AR reduces the incidence of skeletal-related events such as a pathologic fracture, the need for radiation or surgery and the development of cord compression [4].

The risk for MRONJ varies by medication and duration of treatment. MRONJ occurs in approximately 1–9% of patients with advanced cancer [4–14]. The incidence of MRONJ among osteoporotic patients, who mainly receive oral treatment, ranges from 0.001 to 4% [15–21].

One of the most used definitions for MRONJ was specified by the American Association of Oral and Maxillofacial Surgeons (AAOMS). They defined MRONJ as an exposed bone or bone that can be probed through an intraoral or extraoral fistula in the maxillofacial region that persists for more than 8 weeks, without history of radiation therapy to the jaws and occurring in patients with current or previous treatment with AR and/or AA [1].

MRONJ patients are primarily diagnosed with diseases such as breast cancer, prostate cancer, multiple myeloma or osteoporosis that already have a negative impact on the patients´ Quality of Life (QoL) [22–25]. But MRONJ and its symptoms may determine a further decrease of QoL in these patients. Often the patients complain about bad taste and feeding difficulties, pain and discomfort in the mouth [26]. MRONJ can progress to severe forms with involvement of the lower margin and fracture of the mandible, severe maxillary sinusitis, oroantral fistula, orbital abscess, extra-oral fistula, intractable pain and inability to eat, especially when it affects debilitated patients [26].

To date various therapeutic strategies are performed based on the severity of MRONJ, ranging from solely conservative to extensive surgical approaches [27]. Conservative methods include the maintenance of oral hygiene, frequent dental check-ups, antiseptic mouthwash and antibiotic therapy [28]. On the contrary, the surgical approach involves the removal of necrotic bone with subsequent healthy bone margins. Severe cases can require the resection of the affected jaw and the reconstruction with microvascular bone grafts such as the free fibular or iliac crest flap. Experimental therapy includes bone marrow stem cell intralesional transplantation, low-level laser therapy, local platelet-derived growth factor application, hyperbaric oxygen, and tissue grafting [15]. Overall, treatment goals are preservation of quality of life through patient education and reassurance, control of pain, control of secondary infection, prevention of extension of lesion and development of new areas of necrosis [29].

As MRONJ can be challenging to treat, prevention remains crucial to minimize the risk of MRONJ. Patient education regarding oral care methods combined with effective oral health practices are associated with a lower rate of MRONJ [4, 30–33].

However, there is a paucity of studies that report health-related QoL and patient education in MRONJ-Patients and even fewer after surgical treatment. This study aimed to evaluate the health-related QoL in both oncology and osteoporosis patients with MRONJ after surgery and analyzes modalities that could additionally influence QoL such as risk factors and comorbidities. Besides this study examines patients´ perception of knowledge and their awareness regarding the risk of MRONJ and ascertains who educated the patients.

## Methods

### Study design and patient selection

This study was designed as a retrospective observational study among patients with MRONJ who underwent surgical treatment at the Department of Oral and Maxillofacial Surgery, University Hospital Klinikum Rechts der Isar, Technical University of Munich, between May 2016 and August 2020. The study was conducted in accordance with the Declaration of Helsinki and was approved by the Ethics Committee of the Technical University of Munich. All patients provided informed consent to participate.

Eligible patients were identified through a retrospective review of medical records. Inclusion criteria were: (1) diagnosis of cancer or osteoporosis; (2) current or previous use of anti-resorptive or anti-angiogenic medication; (3) confirmed MRONJ diagnosis [29]; (4) no history of head or neck radiation therapy; and (5) having undergone MRONJ-related jaw surgery.

### Data collection and group allocation

All relevant clinical and demographic data were extracted from patient records, including sex, age, primary disease, active principle, duration of treatment, method of administration (intravenous (i.v.), subcutaneous (s.c.) or oral), comorbidities, risk factors, date and number of surgeries and localization (mandible, maxilla, or both).

Patients were then categorized into four postoperative time groups (T1–T4) based on the time interval between surgery and the QoL survey completion:

- **T1**: 2nd– 3rd month after surgery.
- **T2**: 4th– 6th month after surgery.
- **T3**: 7th– 12th month after surgery.
- **T4**: ≥13th month after surgery.

After identifying eligible patients, a single-time QoL questionnaire was sent by mail. This survey assessed patients’ health-related quality of life and their awareness of the risk of MRONJ at their respective postoperative time points. Patients were not followed longitudinally, and each patient participated only once. A total of 154 questionnaires were distributed, and 72 responses were received (response rate: 46.75%). Seven questionnaires were excluded due to incompleteness (*n* = 5) or completion by a family member (*n* = 2), resulting in a final sample of 65 patients for analysis.

### Validation and structure of the questionnaire

The European Organisation for Research and Treatment of Cancer (EORTC) Quality of Life Core Questionnaire (QLQ-C30) has been validated for QoL assessment of patients with types of cancer known to be complicated by MRONJ, namely breast cancer, multiple myeloma, prostate cancer and lung cancer [5, 34]. The Module for Head and Neck Cancer (H&N35) has been validated for MRONJ patients with breast cancer and was also used for MRONJ patients with dysmetabolic bone disease [35, 36]. The results of these studies justify the use of QLQ-C30 and H&N35 for other patients who developed MRONJ.

On average, 12 scales (range 0–18) of the instrument H&N35 were used by the investigators. The scale with the highest percentages of missing values was Sexuality (11.5%) and therefore this scale was not applied in this study [37].

The QoL questionnaire used in this study consisted of four instruments: the 30-item short QLQ-C30, 15 slightly modified items of the H&N35, two questions regarding the MRONJ-specific impact on QoL and three questions assessing the awareness about the adverse effects of antiresorptive and antiangiogenic medication.

### Evaluation of the questionnaire

To not overstrain the participants and risk a smaller amount of responses the original H&N35 was shortened to 15 slightly modified items and named H&N35modified (H&N35m). The response format for both questionnaires was a 4-point Likert scale.

Standard scoring algorithms were applied to produce one global health score, five functional scores (physical, role, cognitive, emotional, and social), nine general symptom scores (fatigue, pain, nausea and vomiting, dyspnea, insomnia, appetite loss, constipation, diarrhea, financial difficulties) and nine oral symptom scores (head and neck (HN) pain, HN swallowing, HN senses, HN dry mouth, HN appearance, HN speech problems, HN social contact, HN weight gain, HN weight loss). All of the scales and single-item measures range in score from 0 to 100. A high score for the global health status represents a high QoL, a high score for a functional scale represents a high level of functioning, whereas a high score for a symptom scale / item represents a high level of symptomatology [38].

To further examine the exclusive impact of MRONJ, patients specified their affliction by the MRONJ on a 4-point Likert scale which was transformed into a score “MRONJ” according to the scoring algorithms by the EORTC [38]. Additionally patients were asked what aspect of their primary disease afflicted them strongly. The question format was designed as a multiple choice question whereby the patient should select all applying answers among pain, nausea, anxiety, consultation of doctors, change of living conditions, physical disabilities, limitations when eating and/or osteonecrosis of the jaw.

### Assessment of patients´ awareness

Two questions assessed patient awareness and knowledge of MRONJ risks, focusing on disease-specific knowledge and perceived adequacy of patient education. To evaluate factual knowledge, patients were asked if they knew which of their medications could be linked to jaw necrosis (Yes/No). Those who answered “Yes” could specify the medication in an open-ended follow-up. This approach captures both objective knowledge (correct identification of drugs) and perceived knowledge (awareness of an association). Similar methodologies have been employed in studies assessing patient awareness of MRONJ risks [39–41]. Patients were also asked if they felt adequately informed about MRONJ at the start of their treatment (Yes/No), followed by an open-ended question on the information source (e.g., physician, dentist, pharmacist). This measure reflects perceived information sufficiency, a key aspect of health literacy and informed decision-making [42].

The questionnaire underwent content validation through expert review by specialists in oral and maxillofacial surgery and oncology. Items were designed to be clear and unambiguous, ensuring face validity and relevance to clinical practice.

### Statistical analysis

For statistical analysis, group means and standard deviations were calculated for each parameter using the Statistical Package for the Social Sciences (SPSS) software, release 26.0, and Microsoft Excel, version 16.41. As the distribution of the various variable scores did not respect the assumptions of normality, the non-parametric Mann-Whitney and Kruskal-Wallis tests were chosen to test the differences between groups. The significance level was set at *p* < 0,05. Secondly, the significance of differences between groups in QLQ-C30 / H&N35m scores when compared to results of other studies was interpreted in terms of moderate (10–20 points), or large (greater than 20) changes in quality of life as defined by Osaba et al. [43]. Whenever possible sex- and age-adjusted reference values were calculated for use in comparisons.

## Results

### Demographic data and overview

65 patients (56.9% female, 43.1% male) with an average age of 74.5 years (range 55–93 years) were included in the study. Most frequently, the primary disease was prostate cancer (*n* = 22), closely followed by breast cancer (*n* = 21), multiple myeloma (*n* = 11) and osteoporosis (*n* = 9). Another patient was diagnosed with lung cancer and one with Non-Hodgkin-Lymphoma.

The most common active principles were zoledronic acid (*n* = 27) and denosumab (*n* = 17). 10 patients changed from bisphosphonates to denosumab during therapy. With an overall mean exposure of 5.7 years (± 4.5) the medication was mainly administered intravenously or subcutaneously (*n* = 52). In total 20 patients received denosumab in an oncological dosis (of those 20 patients 13 patients received only denosumab and 7 patients were administered BP before they started denosumab in an oncological dosis). All information regarding patient characteristics are demonstrated in Tables 1 and 2.

**Table 1:** Patient characteristics

	Characteristics		Distribution (%)
Active principle			
Pamidronic acid	Mean exposure 7.9 years (± 1.6)		2 (3.1%)
Alendronic acid	Mean exposure 13 years		1 (1.5%)
Ibandronic acid	Mean exposure 15.8 years (± 8.2)		3 (4.6%)
Zoledronic acid	Mean exposure 4.3 years (± 3.5)		27 (41.5%)
Risedronic acid	Mean exposure 10 years		1 (1.5%)
Denosumab	Mean exposure 4.0 years (± 3.8)		17 (26.2%)
Bevacicumab	Mean exposure 3.3 years		1 (1.5%)
Change from bisphosphonate to denosumab	Mean exposure 7.5 years (± 4.2)		10 (15.4%)
Unknown			3 (4.6%)
**Risk factors**			
	Hypothyroidism		19 (29.2%)
	Diabetes mellitus		11 (16.9%)
	Rheumatism		2 (3.1%)
	Obesity		4 (6.2%)
	Dental prosthesis / implant		34 (52.3%)
	Preceding surgery in the oral region		28 (43.1%)
	Nicotine abuse		12 (18.5%)
	Alcohol abuse		10 (15.4%)
	Corticosteroid administration		17 (26.2%)
	Osteoporosis (if underlying disease is cancer)		6 (9.2%)
**Primary disease**			
	Prostate Cancer		22 (33.8%)
	Breast Cancer		21 (32.3%)
	Multiple Myeloma		11 (16.9%)
	Osteoporosis		9 (13.8%)
	Lung Cancer		1 (1,5%)
	Non-Hodgkin-Lymphoma		1 (1,5%)
**Point of questioning**			
T1	2nd– 3rd Month		15 (23.1%)
T2	4th– 6th Month		12 (18.5%)
T3	7th– 12th Month		17 (26.2%)
T4	from 13th Month		21 (32.3%)
**Age in years**			
	**Female**	**Male**	**All**
50–59	2 (40.0%)	3 (60.0%)	5 (7.7%)
60–69	12 (70.6%)	5 (29.4%)	17 (26.2%)
ab 70	23 (53.5%)	20 (46.5%)	43 (66.2%)
All	37 (56.9%)	28 (43.1%)	65 (100%)
**Localization of MRONJ**			
	Mandible		44 (67.7%)
	Maxilla		16 (24.6%)
	Both		5 (7.7%)
**Number of surgeries**			
	1		27 (41,5%)
	2		23 (35.4%)
	> 2		13 (20.0%)
	unknown		2 (3,1%)

**Table 2:** Postoperative time group characteristics

	T1	T2	T3	T4	Total
Sex					
female	8	7	10	12	37
male	7	5	7	9	28
EORTC age groups					
until 59	1	0	3	1	5
60–69	4	4	5	4	17
over 70	10	8	9	16	43
Primary disease					
breast cancer	7	3	6	5	21
prostate cancer	5	5	5	7	22
multiple myeloma	2	1	5	3	11
osteoporosis	1	2	1	5	9
lung cancer	0	1	0	0	1
non-hodgkin-lymphoma	0	0	0	1	1
Total	15	12	17	21	65

Two thirds of the MRONJ lesions were located in the mandible (*n* = 44), the maxilla was affected in 16 patients and in 5 patients both jaws showed MRONJ. AAOMS stages were either 2 or 3. In total 23 patients (35.4%) have already had one surgery of the jaw before and 13 patients (20.0%) more than one. Patients were classified into four postoperative groups depending on what point in time after surgery they filled out the questionnaire (T1 = 1–3 months (*n* = 15), T2 = 3–6 months (*n* = 12), T3 = 6–12 months (*n* = 17), T4 = over 12 months (*n* = 21)).

### QoL of the entire patient collective

The global health score with a mean value of 52.6 (± 21.7) for the whole patient collective indicated an impaired general QoL. A direct impact of MRONJ on these patients was detected as well. 10.8% (*n* = 7) of all patients were not at all afflicted by MRONJ, 26.2% (*n* = 17) a little, 32.3% (*n* = 21) quite a bit and 27.7% (*n* = 18) very much. Transformed into a symptom score the mean for all patients was 59.8 (± 32.9). Two third of all patients named the osteonecrosis of the jaw as great affliction of their primary disease (*n* = 42; 64.6%). Affliction remained high among all postoperative groups (T1: 73.3%, T2: 75.0%, T3: 58.8%, T4: 57.1%). 28 (43.1%) patients were afflicted by the change of living conditions. 25 (38.5%) patients declared physical impairment as great affliction and 24 (36.9%) impairments when eating. Pain posed a great affliction for 17 (26.2%), consultation of doctors for 12 (18.5%), anxiety for 11 (16.9%) and nausea for 7 (10.8%) patients.

Oral symptoms, which were measured with the H&N35m questionnaire, were dominated by weight changes, dry mouth, and problems with appearance (see Table 2).

Comparing the QLQ-C30 scores to the sex- and age-adjusted general population norm data for Germany all scores indicated a deterioration of QoL [44] (see Table 3). The means of physical function (63.5 ± 26.4), role function (50.0 ± 36.0), social function (62.8 ± 35.2), fatigue (47.4 ± 31.9), appetite loss (21.5 ± 32.5), constipation (23.6 ± 30.5), financial difficulties (22.2 ± 33.9) and global health/ QoL (52.6 ± 21.7) showed a significant lower score as defined by Osoba et al. [43]. Especially the scores role and social function excelled with a mean difference (md) of more than 20 points [43].

**Table 3:** Means and Standarad deviation of H&N35m-scores entire MRONJ sample

H&N35m	M	SD
HN pain	18,3	22,7
HN swallowing	15,4	17,0
HN senses	18,5	25,5
HN dry mouth	31,8	32,5
HN appearance	27,0	33,3
HN speech problems	21,0	31,5
HN social contact	20,0	33,7
HN weight gain	28,1	45,3
HN weight loss	50,0	50,4
HN MRONJ	59,8	32,9

Qol of patients with cancer or osteoporosis is already decreased, but a comparison to patients that are additionally diagnosed with MRONJ revealed a further decrease of QoL.

Predominantly, it became apparent for role and social functioning. Both for breast cancer, prostate cancer and osteoporosis patients these scores showed moderate to large differences (md ranging from 10.2 to 22.0 for role function and from 10.9 to 26.9 for social function) compared to existing reference values of each primary disease [45–47]. Furthermore breast cancer and osteoporosis patients with MRONJ specified significantly more pain (md = 20.0 and 17.6). Breast cancer and prostate cancer patients with MRONJ also stated greater fatigue (md = 11.6 and 17.7). Financial difficulties affected breast cancer and multiple myeloma patients more strongly (md = 13.4 and 17.2). Complete score values for these comparisons are depicted in Tables 4–7.

**Table 4:** Comparison of MRONJ sample and QLQ-C30 scores of sex- and age-adjusted general population norm data for Germany (M = Mean; sd = standard deviation; md = mean Difference) [44]

	Norm data for Germany	MRONJ-sample		MD
	M	M	SD	
**Global Health/ QoL**	64.1	52.6	21.7	− 11.5
**Functional scales**				
Physical function	75.8	63.5	26.4	− 12.3
Role function	74.7	50.0	36.0	− 24.7
Emotional function	79.3	71.2	25.1	− 8.1
Cognitive function	86.2	78.2	23.6	− 8.0
Social function	85.6	62.8	35.2	− 22.8
**Symptom scales**				
Fatigue	31.8	47.4	31.9	15.6
Nausea/ vomiting	3.9	9.7	20.4	5.8
Pain	33.7	39.2	33.9	5.5
Dyspnea	25.0	29.7	36.2	4.7
Insomnia	31.7	32.3	34.3	0.6
Appetite loss	9.0	21.5	32.5	12.5
Constipation	7.8	23.6	30.5	15.8
Diarrhea	8.4	11.3	22.3	2.9
Financial difficulties	9.5	22.2	33,9	12.7

**Table 5:** Comparison of breast cancer patients with MRONJ and reference scores by the EORTC for patients with metastatic breast cancer (M = Mean; sd = standard deviation; md = mean Difference) [45]

	Breast cancer patients with MRONJ (average age = 71.3 (± 8.7) years)		Patients with metastatic breast cancer (average age = 66.2 (± 15.0) years) [45]		MD
	M	SD	M	SD	
**Global health status**	50,8	17,1	57,6	23,6	− 8,7
**Functional scales**					
Physical function	61.3	20.6	68.5	25.8	− 9.7
Role function	42.9	32.7	64.2	34.3	− 21.6
Emotional function	65,7	31,5	72	22,3	− 4,0
Cognitive function	83,3	25,3	81,7	21,7	1,5
Social function	58,7	40,4	74,9	30,4	− 18,0
**Symptom scales**					
Fatigue	50,3	30,4	39,2	23,1	11,6
Nausea/ vomiting	9,5	17,9	5,8	24,8	5,9
Pain	39,7	29,6	26,8	13,5	20,0
Dyspnea	28,3	34,7	26,8	25,5	4,7
Insomnia	27,0	29,1	34,0	30,6	-1,6
Appetite loss	31,7	38,7	19,9	32,6	4,4
Constipation	22,2	32,2	16,3	27,8	7,1
Diarrhea	6,3	17,1	9,7	28,4	1,1
Financial difficulties	25,4	37,9	13,3	21,7	13,4

**Table 6:** Comparison of prostate cancer patients with MRONJ and reference scores by the EORTC for patients with prostate cancer (2008) (M = Mean; sd = standard deviation; md = mean Difference) [47]

	Prostate cancer patients with MRONJ (average age = 77.5 (± 9.2) years)		Patients with prostate cancer (< 70 years, all stages) [47]		MD
	M	SD	M	SD	
**Global health status**	54,5		67,4	22,2	12,9
**Functional scales**					
Physical function	67,3		73,7	27,4	6,4
Role function	57,6		79,6	30,6	22,0
Emotional function	74,2		79,2	21,6	5,0
Cognitive function	74,2		81,9	21,1	7,7
Social function	70,5		81,4	26,8	10,9
**Symptom scales**					
Fatigue	46,0		28,3	26,8	17,7
Nausea/ vomiting	6,8		5,7	14,6	1,1
Pain	29,5		24,8	30,8	4,7
Dyspnea	31,8		19,5	27,4	12,3
Insomnia	33,3		23,2	29,4	10,1
Appetite loss	16,7		12,1	25,4	4,6
Constipation	27,3		17,3	29,1	10,0
Diarrhea	9,1		8,2	18,8	0,9
Financial difficulties	12,1		6,3	17,8	5,8

**Table 7:** Comparison of multiple myeloma patients with MRONJ and reference scores by the EORTC for patients with multiple myeloma (M = Mean; sd = standard deviation; md = mean Difference) [47]

	Multiple myeloma patients with MRONJ (average age = 73.9 (± 9.6) years)		Multiple myeloma patients (every age, all stages) [47]		MD
	M	SD	M	SD	
**Global health status**	54,55	27,2	55,7	22,8	-1,15
**Functional scales**					
Physical function	67,27	30,2	67,7	23,4	-0,43
Role function	51,52	45,6	60,1	33,4	-8,58
Emotional function	76,52	24,4	71,3	22,7	5,22
Cognitive function	75,76	25,1	78,1	23,8	-2,34
Social function	60,00	41,0	63,2	31,0	-3,20
**Symptom scales**					
Fatigue	39,39	33,8	48,7	26,7	9,31
Nausea/ vomiting	6,06	13,5	10,5	19,2	4,44
Pain	42,42	35,2	47,1	33,6	4,68
Dyspnea	24,24	39,7	26	27,3	1,76
Insomnia	33,33	36,5	28,9	30,6	− 4,43
Appetite loss	12,12	16,8	23,2	30,2	− 11,08
Constipation	12,12	22,5	23,2	29,9	− 11,08
Diarrhea	24,24	33,6	9,6	19,4	14,64
Financial difficulties	33,33	38,5	16,1	26,6	17,23

### Differences between sex

In total women reported worse values on nearly all scales. The greatest differences were displayed in the scores for pain (*p* ≤ 0,021), HN senses (*p* ≤ 0,043) and HN appearance (*p* ≤ 0,009).

### Postoperative time

**Fig. 1:**
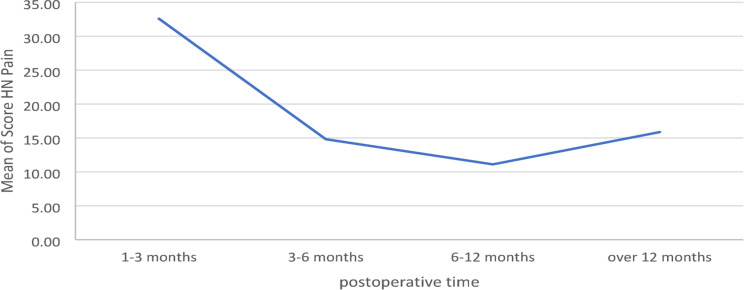
Mean score head and neck pain distributed on postoperative time

Overall Anovatesting between postoperative groups only showed a significant difference for the Score HN Pain (*p* ≤ 0.038) (see Fig. 1). The difference was found between T1 and T3 (Bonferroni correction between T1 and T3 *p* ≤ 0.043).

Dividing the patient collective into two postoperative groups, before and after a certain postoperative time the greatest differences were found at the threshold of 8 months.

Patients in the first 8 months after surgery were significantly more afflicted by MRONJ (*p* ≤ 0.026), had more problems regarding swallowing (*p* ≤ 0.022) and suffered from more head and neck pain (*p* ≤ 0.040).

Another threshold that showed great differences between before and after this point in time was detected at 11 months. Patients further than 11 months after surgery showed worse cognitive function (*p* ≤ 0.008), more financial difficulties (*p* ≤ 0.019), more problems with their appearance (*p* ≤ 0.024), and more trouble with social contact (*p* ≤ 0.049).

### Associated factors

One factor that significantly affected QoL of MRONJ patients was nicotine abuse. Almost all determined scores were diminished compared to patients without a known history of nicotine abuse. QoL was especially influenced by financial difficulties (*p* ≤ 0.030) and problems regarding swallowing (*p* ≤ 0.024).

Patients with a dental implant or denture specified more symptoms regarding swallowing (*p* ≤ 0.050, differences in T2 and T3). On the other hand they expressed less symptoms regarding insomnia (*p* ≤ 0.002). 52.9% (*n* = 18) of the patients with a dental implant or prosthesis reported impairments when eating compared to 19.4% (*n* = 6) of the patients without a dental implant or prosthesis. When being asked what they are most afflicted by a total of 5 patients specifically named missing teeth and inability to wear dental prosthesis or speech problems in the field “other”.

Another factor that seemed to compromise QoL was rheumatism. MRONJ patients with rheumatism showed worse scores for HN Swallowing (*p* ≤ 0.023), HN Speech (*p* ≤ 0.016) and HN Social contact (*p* ≤ 0.037). QoL of Patients with hypothyroidism was deteriorated regarding their appearance (*p* ≤ 0.021). Patients with diabetes mellitus had more trouble with social contact (*p* ≤ 0.017). Patients with obesity reported less head and neck pain (*p* ≤ 0.044) and more weight gain (*p* ≤ 0.001).

Pre-operated patients remarked more problems with swallowing (*p* ≤ 0.022), speech (*p* ≤ 0.000) and weight loss (*p* ≤ 0.019). Such impairments were observed during all postoperative points in time. In the first 6 months after surgery pre-operated patients complained more about head and neck pain (*p* ≤ 0.021), speech problems (*p* ≤ 0.003) and weight loss (*p* ≤ 0.038) (1 surgery *n* = 17; ≥2 surgeries *n* = 20). Above these 6 months they were more adversely affected by general pain (*p* ≤ 0.011), speech problems (*p* ≤ 0.039) and swallowing (*p* ≤ 0.042) (1 surgery *n* = 10; ≥2 surgeries *n* = 16).

### Awareness

A third of all patients (*n* = 21) were not able to name the medication causing their MRONJ. More than half the patients (*n* = 35) did not feel adequately informed about MRONJ before beginning AR treatment.

Those patients that felt adequately informed (*n* = 29) were mainly informed at the department of oral and maxillofacial surgery at the University Hospital “Rechts der Isar” (*n* = 17) or by an oral and maxillofacial surgent at an external hospital (*n* = 2). 10 patients named their dentist or orthodontist as informer and 3 their oncologist.

The knowledge about the medication did not differ between sex. Yet, only 35.7% (*n* = 10) of men but 69.4% (*n* = 25) of women did not feel adequately informed.

Breast cancer, prostate cancer and multiple myeloma patients could not name the medication on an equal level (33.3% (*n* = 7); 36.4 (*n* = 8); 36.4% (*n* = 4). Only osteoporosis patients were better at naming the right medication, only 1 patient (16.7%) was not able to. Primary diseases also had an impact on the adequate information of patients. 66.7% (*n* = 14) of breast cancer patients, 62.5% (*n* = 5) of osteoporosis patients, 54.5% (*n* = 6) of multiple myeloma patients, 36.4% (*n* = 8) of prostate cancer patients and both the lung cancer and the NHL patient did not feel adequately informed about MRONJ.

Awareness had an impact on QoL of patients with MRONJ. The patients that felt adequately informed were less afflicted by head and neck pain (*p* ≤ 0.004) and experienced less problems with swallowing (*p* ≤ 0.049). They also reported better emotional (*p* ≤ 0.026) and social function (*p* ≤ 0.038). This could also be observed on varying postoperative points in time.

Within T1 patients who felt adequately informed revealed better social function (*p* ≤ 0.002, after Bonferroni Correction $\:{\alpha\:}_{adj}=\text{0,0125}$).

If patients were both capable auf naming the medication and secondarily felt adequately informed (*n* = 22) they indicated better emotional functioning (*p* ≤ 0.003, after Bonferroni Correction $\:{\alpha\:}_{adj}=\text{0,0125}$).

In addition to a better QoL patients who felt adequately informed were less frequently operated on the MRONJ (*p* ≤ 0.034). Patients who felt adequately informed underwent a mean of 1.6 (± 0.9) MRONJ surgeries (median = 1), whereas patients who did not feel adequately informed had a mean of 2.2 (± 1.2) MRONJ surgeries (median = 2).

## Discussion

### Study characteristics and limitations

MRONJ is a debilitating condition often combined with pain that can adversely affect a wide range of general and oral health-related QoL domains. Above all this is important due to the nature of MRONJ as it concerns oncologic and osteoporosis patients who already have a decreased QoL. Measuring patient-reported outcome is essential to allow evaluation of the impact of the disease and its associated factors on patients, and to gather evidence-based information of the effectiveness of an intervention.

The patient collective was in keeping with the common epidemiology. MRONJ predominantly affects older patients and the mean age in the majority of the published studies is over 60 [48, 49]. In total there are slightly more women included in this study (56.9%), but as osteoporosis and breast cancer are the most frequent reasons for the prescription of BP, these results are not surprising [48]. The mean duration of exposure with 5.7 years was similar to other studies (5.3 years [49]). The localization of MRONJ with occurring twice as often in the mandible than in the maxilla and rarely in both jaws is also consistent with many other previous publications [49–51].

This study has some limitations that need to be acknowledged. The sample size is rather small which is owed to the low incidence of MRONJ [52] and consists of a very heterogeneous population. This makes it difficult to draw general conclusions as the classification of patients by active principle, method of administration and primary disease quickly results in small numbers.

The chosen study model with mailing of the questionnaires can reach a large number of patients, but is strongly dependent on the compliance of the participants. This is reflected in the response rate of 46.75%. However, the mortality rate of the potential participants, which is likely to be quite high due to the underlying oncological disease and the advanced age, also has a major influence. When a bone metastasis occurs, the disease is usually already in an advanced stage. The median survival time of patients with bone metastases is 9 months. Only 4.5% of those affected survive longer than 24 months, while more than 50% lose their lives within the first 6 months [53].

The aim of the study was to access life quality after surgery and to evaluate the awareness of MRONJ. Further, the study was carried out to evaluate the created questionnaire. With the findings of this study, further studies to compare the pre- and postoperative values in MRONJ could be started.

### General QoL

As expected the Global Health/QoL and other aspects of general QoL were significantly deteriorated which was confirmed through the comparison to the German normative population. Particularly role and social function were adversely affected. The sensitivity for role function in MRONJ patients has already been examined in prior studies [35]. A deteriorated social function means that the patient´s physical condition and medical treatment interfere with his family life and with his gathering or social activities with other people. Apparently MRONJ patients feel more impaired in their everyday life.

As the primary disease depicts a confounding factor when measuring QoL of MRONJ patients we compared the results to patients solely diagnosed with osteoporosis or cancer. The significant lower values suggest that MRONJ has an additional negative impact on QoL. The particular affection of role and social function was also noticed in this comparison.

### Oral QoL

The oral symptoms reported in this study are similar to the results of other MRONJ studies that name head and neck pain, difficulty with eating and speaking, troubles with social contact, problems with teeth and a dry mouth as most common symptoms [48].

Kyrgidis et al. analyzed the QoL of MRONJ patients with metastatic breast cancer with or without MRONJ and patients with cancer of the oral cavity group [35]. For the MRONJ patients they reported lower role function, more head and neck pain, worse problems with senses, speech, social eating and more troubles with social contact. Females in our study reported similar role function, head and neck pain, speech problems and troubles with social contact compared to the MRONJ group of Kyrgidis et al. The score for social function was worse and the score for problems with senses was better than Kyrgidis et al. Further, question 3 and 6 of our questionnaire were modified, since they were based on our clinical experience, patients are telling us these items during the consultations. Nevertheless, the readers should be aware, that these two questions have a lower reliability than the other questions. The H&N35m questionnaire also includes a question with only two response options: “What bothers you most about your underlying condition?” For the second question, patients could select multiple options from the following: pain, nausea, anxiety, doctor visits, changes in life circumstances, physical disabilities, eating restrictions, jaw necrosis, and other (with a text field for a free response). This question offers therefore also lower reliability. We asked it, since it is an important question and sheds light to the patients biggest fears and problems.

### Financial difficulties, eating impairments and change of living conditions

When being asked what aspect of their primary disease poses the greatest affliction, two thirds of all patients named the MRONJ. This reveals the actual severity of this disease and its effect on patients´ QoL. Another major factor was the affliction by the change of living conditions. A number of patients declared financial difficulties caused by their physical condition and medical treatment which could lead to a change of living conditions. Besides over a third of the patients were physically impaired or impaired when eating which could result in trouble with social contact, problems with the appearance, speech problems and above all a change of living conditions.

### Postoperative time

As expected patients in T1 reported the worst QoL results. Surgery for MRONJ affects many structures that are necessary for adequate articulation, chewing and swallowing [54, 55]. In particular patients in T1 felt most impaired by speech problems and inability to gain weight, although not statistically significant.

Another often reported symptom was “pain.” Even though some patients did not feel any pain the majority suffered from general pain and especially from pain in the head and neck region. As a result, we observed high scores in “swallowing” which include problems with chewing and swallowing liquids and soft or solid food. The scores were high in one to three months after surgery, then decreased and stayed on an equal level. Nevertheless, the score swallowing showed no statistical differences between postoperative groups in an Anova analysis. Moll et al. reported that compared to before surgery “pain” scores decreased and so did “swallowing” problems. In consequence of less eating problems “weight loss” also showed lower scores with higher scores in “weight gain” [49]. These findings correlate with our observations.

Whereas the first 8 months after surgery seem to be dominated by mainly physical complaints like problems regarding swallowing and head and neck pain and above all a high general affliction with MRONJ, after 11 months the adverse affection switches to social domains such as financial difficulties, problems with the own appearance and troubles with social contact. These could be interpreted as long-term consequences of longsome treatment and isolation from society.

Many patients in this study had trouble with their appearance which could be caused by swelling, redness, inability to open the mouth and bad breath in the first months after surgery. However, we noticed even worse scores after twelve months after surgery, especially for women. Possible reasons could be older age, a higher number of relapses or it might be a long-term effect after surgery as it sometimes becomes visible from the outside when bone was removed or replaced.

The symptoms MRONJ patients complain about might change over time and although the patients with more than 6 months after surgery are less frequently afflicted by MRONJ it still remains the majority with over 57% even in T4.

The question whether surgery of the MORNJ increases the QoL is still subject of debates. Several studies state that patients profit from surgical intervention shown through a full mucosal healing rate of 85% in MRONJ stage III patients [56] and found significant improvement of QoL over postoperative time for the following scores: “swallowing” (*p* = 0.007), “opening mouth” (*p* = 0.045), “painkiller” (0.005), “weight loss” (0.004), “pain” (*p* = 0.001), “trouble with social eating” (*p* = 0.001), “trouble with social contact” (*p* = 0.001), and “teeth” (*p* = 0.001) [49].

### Associated factors

In this sample, the factors that are associated with deteriorated score values are both sociodemographic (age, female sex (although this might be due to the underlying disease)) and clinical (primary disease, nicotine abuse, rheumatism, hypothyroidism, diabetes mellitus and wearing of dentures). The deteriorated scores show how known risk factors of MRONJ can additionally influence the QoL of patients.

Nicotine abuse is a known risk factor associated with MRONJ development and the risk of MRONJ increases with each pack year of smoking [57]. Nicotine abuse has been linked with effects to all organs in the human body. In the oral cavity, nicotine abuse impairs wound healing and worsens periodontal disease [58–60]. We observed that nicotine abuse is also associated with a worse QoL such as more financial difficulties and problems with swallowing. So far there are no other studies about nicotine abuse and QoL of MRONJ patients available, but similar observations were made for head and neck cancer patients. Smokers show deteriorated scores on all QLQ-C30 scales, in particular social function, dyspnea, pain and financial difficulties. Oral health-related QoL of smokers is impaired by head and neck pain, troubles with social eating, problems with teeth and mouth opening, less saliva and more sexual problems [61]. Besides smokers show a higher risk of MRONJ relapse that lasts longer than 6 months meaning they remain with exposed bone [59]. Prevention of smoking onset and support for cessation of smoking could contribute to an improved oral health status and result in a better QoL.

Surgical intervention such as dental extraction was performed in 43.1% of the patients before MRONJ first occurred. Studies have shown that this is a common predisposing event [5, 62, 63]. Then again Otto et al. has described that not the dental extraction itself depicts the trigger for the development of MRONJ but rather a prevailing infectious condition in the bone may increase the risk. By performing dental extractions according to established guidelines which include perioperative antibiotic prophylaxis, atraumatic surgery, smoothening of sharp edges of the bone, and saliva tight wound closure, dental extractions can be performed in a safe way [64]. As most dental extractions were not performed at our department, we cannot confirm whether these suggestions were obtained.

MRONJ patients who usually wear a denture or have a dental implant specify more problems regarding swallowing and impairments when eating. When diagnosed with MRONJ patients are recommended not to wear their denture due to pressure marks which could enhance the development of MRONJ. This could be one reason for more difficulties in swallowing. Moll et al. mentioned how in some cases it appeared that the event of getting new prosthesis had an essential impact on how patients experienced their current situation. The question when to get new dental prosthesis was frequently asked. Depending on the location and the healing progress, they suggested to wait at least 6 weeks after surgery [49].

Despite a continuous development of treatment protocols the relapse rate for MRONJ remains high. Varoni et al. observed complete healing for 88.5% of all patients one month after surgery, but 30.4% showed recurrence after a mean period of 7.29 ± 3.45 months [65]. Moll et al. [49] described recurrences for 25.6% of all patients in the first 6 months with two third occurring in the first 6 weeks. Hence, it is not surprising that 55.4% of all patients in this study have had more than one surgery. In contrast to Moll et al. who could not find any QoL differences between patients with or without recurrence, we detected a significantly deteriorated QoL for patients with more than one surgery. In the comparison to patients with only one surgery the foremost symptoms were pain and problems regarding swallowing and speech, even after more than 6 months after the last surgery. This emphasizes the need for a further development of treatment strategies, prevention and reduction of possible risk factors.

### Awareness

MRONJ results in a worse QoL for osteoporosis and cancer patients. Yet the overall benefit-risk balance of denosumab an bisphosphonates is positive [66]. The key to avoid MRONJ therefore lies within prevention both before, during and after treatment. This includes informing patients about maintaining oral hygiene, avoidance of unnecessary dental surgery and dental infections, full dental assessment and education on key signs and symptoms of MRONJ. Regular dental check-ups and improved oral hygiene can reduce the incidence of MRONJ [32, 33]. In order to ensure the compliance with preventive measures, the patients need to be aware of the potential risk of developing MRONJ.

This study shows that the majority of patients did not feel well enough informed about the risk of developing MRONJ. These results are similar to those from Al Abdullateef et al. where only 33.82% of the patients were aware [67]. In another study only 12.4% of participants were aware about the risk of MRONJ following BP use, and only one third of them has received information from their prescribing physicians [68].

Supanumbar et al. described in 2024 that approximately 60% of physicians informed patients of the MRONJ risk before prescribing antiresorptive drugs, and 30% inquired about patients’ oral symptoms at the follow up visit. Overall, 44% of physicians advised patients to receive oral health care; the most common reason for not advising this was that respondents did not conside themselves to be adequately knowledgeable to detect oral health problems [69]. Another study stated, that among 192 medical doctors, 21.9% had not heard of the disease. Only 8.9% correctly answered all questions testing BRONJ knowledge. Dental referrals made by medical doctors were implemented in less than 30% of the total patients. Oncology specialists most often recognized the necessity of dental referrals followed in decreasing order by endocrinology, rheumatology, family medicine, and orthopedic specialists [70]. Another important study, was done by Sturrock et al., the study group evaluated limited knowledge and awareness of MRONJ and saw it as an effect due to the complex medical histories of patients, that practioners often over looked the advice related to the risk and prevention of MRONJ [71].

Compared to other primary diseases osteoporosis patients were better at naming the right medication. On average osteoporosis patients received AR for a longer time period than cancer patients. Therefore they paid more frequently visits to the prescribing doctor which could result in a better patient education.

For further studies to improve assessment of awareness and information, more answer options and diverse questions should be used, to gain higher reliability for the two questions as a construct for awareness and information. As we recognized these results regarding awareness and information, we did not expect before, we conclude that future studies nedd to bring more light to this topic with extended versions of these kind of questions.

The importance of adequate patient education is strongly emphasized by the number of required surgeries with a median of one surgery for aware patients compared to a median of two surgeries for unaware patients. As patients with only one surgery show a better QoL, it is only logical that aware patients also report a better QoL. Aware MRONJ patients reported better QoL in social dimensions (emotional and social function). Most importantly they had better emotional function which implies less tense, depressed or irritable feelings and less worrying. Patient education not only helps in terms of preventing MRONJ and its symptoms but also serves the emotional immunity during and after the disease.

There are many possible reasons for a better QoL of aware patients. On the one hand MRONJ can be detected earlier for example due to regular dental examinations and therefore treated at an earlier stage. On the other hand aware patients most likely show better compliance with maintaining good oral hygiene and avoiding risk factors. Yet, no significant differences in terms of physical domains between aware and unaware patients could be determined in this study. Further studies with a greater number of patients are necessary.

Only a third of the patients that felt adequately informed named their dentist and only a tenth their oncologist as an informer about MRONJ. The rest was informed by an oral and maxillofacial surgent. To achieve the best outcome for patients with MRONJ, a multidisciplinary approach is crucial and should involve dentists, nurses, primary care physicians, oncologists, oral and maxillofacial surgeons and the patient. Both patients and healthcare professionals need to remain vigilant for the signs and symptoms of MRONJ throughout treatment. MRONJ can be managed effectively with respect to symptom control and QoL, and the risk of developing the condition can be substantially reduced if preventive measures are taken [31].

MRONJ significantly influences both general and oral-health-related QoL of patients with osteoporosis or cancer. With the impending introduction of more antiresorptive and antiangiogenic principles into routine clinical practice, rapid demographic change and raising longevity of cancer patients, MRONJ and its adverse affection of QoL will gain even more relevance in the future. Validated questionnaires such as the H&N35 should be administered regularly to every AR receiving patient. Increasing awareness of head and neck symptoms in osteoporosis and cancer patients might help diagnose those who develop MRONJ earlier. So far no specific instrument for measuring QoL of MRONJ patients is available. Further studies are necessary to validate a questionnaire particularly targeted on QoL of MRONJ patients. This would improve the comparability of the collected data.

The current lack of awareness is alarming. Overall, a multidisciplinary approach to the prevention and management of MORNJ should be adopted. Raising the awareness regarding signs and symptoms, oral hygiene and dental assessment of both health care professionals and patients must become an inherent part of clinical practice guidelines.

Nevertheless to these findings the limitations of the study are at one hand the lack of the assessment of the HRQoL before surgery and at the other hand the assessment of only one timepoint of every patient. These limitations made conclusions not possible based on HRQoL changes before and after surgery. For future study trials these limitations should be addressed as important facts focusing HRQoL in MRONJ.

## Data Availability

No datasets were generated or analysed during the current study.

## Abbreviations

MRONJ: Medication-Related Osteonecrosis of the Jaw
QoL: Quality of Life
EORTC: European Organisation for Research and Treatment of Cancer
QLQ-C30: Quality of Life Core Questionnaire
H&N35: Module for Head and Neck Cancer
H&N35m: Module for Head and Neck Cancer modified
AR: Antiresorptive agents
AA: Antiangiogenic agents
AAOMS: American Association of Oral and Maxillofacial Surgeons
i.v.: Intravenous
s.c.: Subcutaneous
HN: Head and neck
SPSS: Statistical Package for the Social Sciences
MD: Mean Difference
M: Mean
SD: Standard Deviation
f: Female
m: Male
